# IMPLEMENTATION OF THE COMMUNITY HEALTHCHOICES PROGRAM

**DOI:** 10.1093/geroni/igac059.564

**Published:** 2022-12-20

**Authors:** Teresa Beigay

**Affiliations:** University of Pittsburgh, Pittsburgh, Pennsylvania, United States

## Abstract

The Community HealthChoices program was implemented in three phases, starting in 2018 in the SouthWest region of Pennsylvania, followed by the SouthEast in 2019 and the remainder of the state in 2020. Interviews were conducted with participants in each region during the first several months of each implementation. Focus groups were conducted in the months immediately following each phase. We reviewed all participant materials and public education events in each region. The percentage of participants who reported having received information about the new program increased in each phase. This is consistent with our observation that more public education events were held in 2018 and 2019 than in 2017. However, satisfaction with information did not improve over time. Focus groups revealed a mixed picture of successes (new benefit cards were received and claims were paid) and challenges (providers were not aware of the program; selecting primary care providers was confusing).

